# Quality of Care Is Improved by Rapid Short Incubation MALDI-ToF Identification from Blood Cultures as Measured by Reduced Length of Stay and Patient Outcomes as Part of a Multi-Disciplinary Approach to Bacteremia in Pediatric Patients

**DOI:** 10.1371/journal.pone.0160618

**Published:** 2016-08-11

**Authors:** Johannes A. Delport, Arend Strikwerda, Amanda Armstrong, David Schaus, Michael John

**Affiliations:** 1 Division of Microbiology, Pathology and Laboratory Medicine, London Health Sciences Centre, London, Ontario, Canada; 2 Schulich School of Medicine and Dentistry, Western University, London, Ontario, Canada; 3 Department of Microbiology and Immunology, Schulich School of Medicine and Dentistry, Western University, London, Ontario, Canada; Universita degli Studi di Parma, ITALY

## Abstract

Sepsis has seen an incremental increase in cases of about 13% annually in the USA and accounts for approximately 4400 deaths among pediatric patients. Early identification of the specific pathogen allows the clinician to ensure that the antibiotic coverage is optimal, an intervention that has been shown to improve patient outcomes in sepsis. Our study’s objective was to assess the impact of a rapid Bruker MALDI-Tof identification protocol on pediatric sepsis cases by assessing various indicators. We assessed the quality of care by measuring the following indicators; time to identification of the pathogen, initiation of the most appropriate antibiotic, length of stay (LOS) in hospital and patient outcomes, using a retrospective review over three consecutive years. In total 92 pediatric patients, similar in age and gender distributions were assessed; 37 in 2012, 33 in 2013 and 22 in 2014. The introduction of MALDI-TOF identification in 2013 led to a significant decrease in time to identify a pathogen by 21.03 hours (p = 1.95E-05). A short incubation MALDI-TOF identification protocol in 2014 further reduced time to identification by 17.75 hours (p = 2.48E-3). Overall in 2014 this led to a trend to earlier optimization of antibiotics by 20.2 hours (p = 0.14) and a reduction in length of stay after the implementation of MALDI-ToF identification in 2013 of 3.07 days and a further reduction of 8.92 days after the introduction of the rapid short incubation identification protocol using MALDI-Tof in 2014 (P = 0.12). By evaluating the subgroup of patients where antibiotics were changed, our study confirmed that patients received appropriate therapy 48.8% (20.2 hours) earlier compared to conventional methods leading to a decrease in length of stay of 23.65 days after the implementation of MALDI-ToF identification and a further reduction of 9.82 days in 2014 compared to 2012 (p = 0.02). In 2014 outcomes between the patients needing a change in their antibiotic compared to the patients where the empirical therapy was considered to be optimal were similar with respect to length of stay; 13.04 and 10.93 days (p = 0.34). In the 2012 group there was a significant increase in the length of stay in the group needing change in excess of 30 days (p = 0.02) compared to the group where empirical therapy was considered to be optimal, this clearly showed an improvement in the quality of care received after the rapid identification was instituted in 2014. The 2012 group had a four times overall increased sepsis associated mortality risk compared to the 2014 group and when empirical antibiotics needed to be optimized this risk was 7 times compared to the 2014 group. We conclude that rapid identification of bacterial pathogens in pediatric blood cultures with a rapid incubation MALDI-TOF identification protocol plays an important role in improving quality of care as part of a multidisciplinary approach to pediatric bacteremia and sepsis.

## Introduction

The introduction of Matrix-assisted laser desorption ionization-time of flight (MALDI-TOF) mass spectrometry has significantly shortened the clinical microbiology laboratory’s time to identification of pathogens [1]. This, along with short incubation culture from blood samples means the laboratory is able to provide clinicians with reliable identification of pathogens up to a day earlier [1]. Although empiric therapy for suspected sepsis is instituted as soon as possible, early identification of the specific pathogen allows the clinician to ensure that the antibiotic coverage is appropriate and optimal, an intervention that has been shown to improve patient outcomes in sepsis [2, 3, 4].

This study was carried out to see whether earlier identification and reporting of organisms in pediatric sepsis influenced timing of appropriate antibiotic therapy and patient outcomes.

## Study Design

A retrospective chart review of pediatric patients with positive blood cultures over the same 3-month period (January to end of March) in three consecutive years, 2012 to 2014 was performed. The aim was to assess the impact the introduction MALDI-ToF identification (MID) (2013) and rapid short incubation MALDI-Tof identification protocol (SIMI) (2014) had on patient care compared to conventional identification (CID) (2012). The data obtained from our Cerner laboratory information system was time to identification of the organism, time to initiation of the most appropriate antibiotic, and length of stay in hospital. The primary objective was to demonstrate an improvement in patient care as demonstrated by improved outcomes and reduced length of stay.

## Materials and Methods

### Ethics

The study was submitted as part of a quality improvement initiative to the Office of Research Ethics, Western University, London, Ontario (Research and Ethics number 106659). In accordance with the Tri-Council Policy Statement 2: Ethical Conduct of Research Involving Humans, Article 2.5 ethics approval was waived as the study fulfilled the criteria of a quality improvement study and therefor did not constitute research and as such no written permission was required (S1 Waiver). The patient demographics were collected from the Laboratory Information System as part of the Microbiology Quality Improvement Program. All data were de-identified by the primary investigator prior to analysis and are stored in a secure location. The authors were part of the circle-of-care but were not the primary attending physician.

### Data Collection

Positive blood cultures from the first of January to the end of March of 2012, 2013 and 2014 were selected from pediatric patients between the ages of six months and eighteen years and a retrospective chart review performed to assess the impact of earlier identification by MALDI-ToF (2013) and rapid short incubation identification protocol (2014). We measured the time to identification of the organism, initiation of the most appropriate antibiotic, the length of stay in hospital and collected data on patient demographics to assess quality indicators. A subgroup of patients were identified where the initial antibiotic coverage was changed, based on the identification of the pathogen. End user response was assessed according to the appropriate antibiotic selected as set out in the London Health Sciences formulary and antibiogram.

### Blood Cultures

Blood culture bottles were collected, incubated and processed according to the manufacturer’s protocols (VersaTrek, TREK diagnostics, Thermo Fisher Scientific Inc., USA). All blood cultures that were identified as positive received a Gram and were processed according to the methods below.

#### Pathogen Identification

In 2012 positive blood cultures were plated on Columbia 5% sheep blood agar, MacConkey agar and chocolate agar (Oxoid, Canada) and incubated in appropriate atmospheres. Plates were assessed for discernible colonial morphology after 8 and 18 hours of incubation. Identification was based on standard phenotypic, biochemical and or Vitek 2 (bioMérieux, Canada) automated methods. In 2013 identification was performed using the Bruker MALDI-ToF BioTyper (Bruker Daltonics, Germany) as per recommendations of the manufacturer, following our standard culture protocol. The short incubation protocol on Columbia 5% sheep blood agar was implemented and identification was performed on the Bruker MALDI-ToF BioTyper in 2014. The short incubation protocol differed from our standard procedure in that positive blood cultures were plated on 5% sheep blood agar and the surface of the agar plates swabbed after 3 hours of incubation in 5% CO_2_ at 35°C. The product was then processed for pathogen detection and identification with the Bruker MALDI-ToF BioTyper.

#### Bruker MALDI-ToF BioTyper

The test organisms were applied to each target spot as a thin monolayer using the direct colony transfer method according to the manufacturer’s recommendation. The target spot was allowed to dry and 1uL of 70% formic acid was added, followed by 1uL of matrix (Bruker, HCCA #8255344) and the target plate allowed to dry at room temperature.

#### Identification Criteria for the Bruker MALDI-ToF BioTyper

The identification of the isolates were performed on a Microflex LT instrument (Bruker Daltonics, Germany) with FlexControl (version 3.0) software (Bruker Daltonics, Germany) for the automatic acquisition of mass spectra in the linear positive mode within a range of 2 to 20 kDa. Automated analysis of the raw spectral data was performed by the MALDI BioTyper automation (version 2.0) software (Bruker Daltonics, Germany). Identification criteria were followed as specified by the manufacturer.

### Statistical Analysis

Baseline data were summarized and tested for differences. Analysis of variance was used to assess differences in age, gender and comorbidity, all categorical variables were assessed for statistical significance with the Mann-Whitney *U* test, taking into account the small and diverse study population (S1 Table).

## Results

In total 92 pediatric patients were assessed, 37 in 2012, 33 in 2013 after implementation of MALDI-ToF identification and 22 in 2014 after addition of the rapid short incubation identification protocol. The groups were similar in age (p = 0.145) and gender distribution (p = 0.09) (Table 1). Although the patient group in 2014 had a slightly higher mean age than the other groups this was not clinically significant. The comorbidities were similar among the groups (p = 0.35). The 2014 group had two patients with comorbidities associated with long hospital admissions while the 2013 and 2012 groups respectively had two and one such associated comorbidities. The three patients in the 2013 and 2014 groups with complicated long-term comorbidities were all due to hematological malignancies and or chronic pulmonary failure not associated with an infectious agent, while the patient in the 2012 group had a cerebrovascular incident complicating hospital stay. Two patients in the 2014 group had documented initiation of antibiotics prior to hospital admission, both of these patients antibiotics were changed and optimized after identification of the pathogen. In the 2012 group one patient was started on antibiotics prior to hospital admission, empirical antibiotic therapy was considered to be optimal coverage for the pathogen and treatment was not adjusted after identification. One death occurred in the 2012 group that was attributable to sepsis; allthough one death occurred in the 2014 group this death was attributed to chronic unresolvable respiratory failure. All data was measured from the time the patient was admitted with the exception of information relating to blood culture pathogen identification, which was measured from the time the blood culture was collected.

**Table 1:** Summary of the Results Obtained with the Retrospective Analysis of the Three Pediatric Patient Groups.

	Conventional identification (2012)	MALDI-ToF identification (2013)	Rapid short incubation (2014)
**Average age in months, (range)**	17.9, (6.2–48.9)	18.9, (9–42.9)	23.1, (7.2–52.6)
**Average comorbidities per patient, (range)**	0.8, (0–2)	1.0, (0–5)	0.8, (0–4)
**Male gender, number, (%)**	25, (67.57)	26, (78.79)	10, (45.45)
**Female gender, number, (%)**	12, (32.43)	13, (21.21)	12, (54.55)
**Average time to initiate antibiotics, (range)**	14.5, (-2.65–34.71)	14.38, (-12.08–65.68)	9.12, (-0.57–24.65)
**Average time to Gram stain result, (range)**	29.62, (9.91–91.78)	27.29, (14.41–52.28)	25.21, (6.46–63.88)
**Average time to initial identification, (range)**	79.52, (23.95–140.85)	58.49, (24.85–213.31)	40.73, (14.55–154.27)
**Average time to final identification, (range)**	87.23, (23.95–146.81)	75.70, (28.18–213.31)	70.53, (14.55–154.27)
**Average time to antibiotic change, (range)**	78.33, (51.25–106)	68.76, (60.33–115.5)	58.13, (122.08–127.42)
**Average length of hospital stay in days, (range)**	23.81, (0.21–151.40)	21.71, (0.08–112.72)	12.28, (0.20–47.52)
**Average LOS in correct empirical therapy group in days, (range)**	20.79, (0.12–106.33)	22.15, (0.28–112.72)	13.04, (0.20–47.53)
**Average LOS in antibiotic change group in days, (range)**	44.40, (3.64–151.4)	20.75, (2.60–47.45)	10.94, (2.87–17.68)

The changes that occurred in the three different groups are summarized in Table 2. The results reflect that changes occurred in the diagnostic and clinical management of the groups in the three consecutive years. The components considered to be most instrumental in instituting effective empirical antibiotic treatment were that the time to initiation of antibiotics and Gram stain were reduced by 36% (5.25 hours) and 15% (4.41 hours), although both were not statistically significant. The initial identification of the pathogen was reduced by 48.6% (38.78 hours, p = 7.46 E-07) and the antibiotic therapy was overall optimized 25.8% (20.2 hours, p = 0.14) earlier compared to 2012. The overall length of stay is reduced by 49.4% or 11.99 days (p = 0.10) after the rapid identification protocol was introduced in 2014, compared to conventional identification in 2012 (Table 2).

**Table 2:** Time Difference Between the Groups After Implementation of MALDI-ToF and Short-Incubation Identification.

	Conventional identification vs. MALDI-TOF identification (2012–2013)		MALDI-TOF identification vs. SIMI (2013–2014)		SIMI vs Conventional identification (2012–2014)	
Average change	Δhours	P, (median_2012,2013_; *U*)	Δhours	P, (median_2013,2014_; *U)*	Δhours	P, (median_2012,2014_; *U)*
**Time to initiate antibiotics**	0.13	0.43, (8.23, 10.05; 447)	5.25	0.19, (10.05, 6.57; 423)	5.38	0.14, (8.23, 6.57; 161)
**Time to Gram stain result**	2.33	0.22, (26.94, 22.23; 677)	2.08	0.22, (22.23, 22.15; 392)	4.41	0.14 (26.94, 22.15; 403)
**Time to initial identification**	21.03	1.95E-05, (70.17, 47.47; 291)	17.75	2.48E-3, (47.47, 32.93; 230)	38.78	7.46E-07, (70.17, 32.93; 128)
**Time to final identification**	11.54	0.01, (84.67, 70.3; 495)	5.17	0.4, (70.3, 68.13; 427)	16.71	0.03, (84.67, 68.13; 331.5)
**Time to antibiotic change**	9.56	0.19, (66.98, 79.5; 40)	10.64	0.03, (79.5, 49.17; 35)_	20.2	0.14, (66.98, 49.17; 27)
**Length of hospital stay in days, (hours)**	3.07 (73.68)	0.50, (293.23, 280.00; 659)	8.92 (214.08)	0.14, (280, 273.62; 301)	11.99 (288.00)	0.10, (293.23, 273.62; 326)

The impact on patient treatment are summarized to show how the implementation of the MALDI-ToF identification and the short incubation protocol effected different stages in the diagnostic and identification process of positive blood cultures.

**Fig 1:**
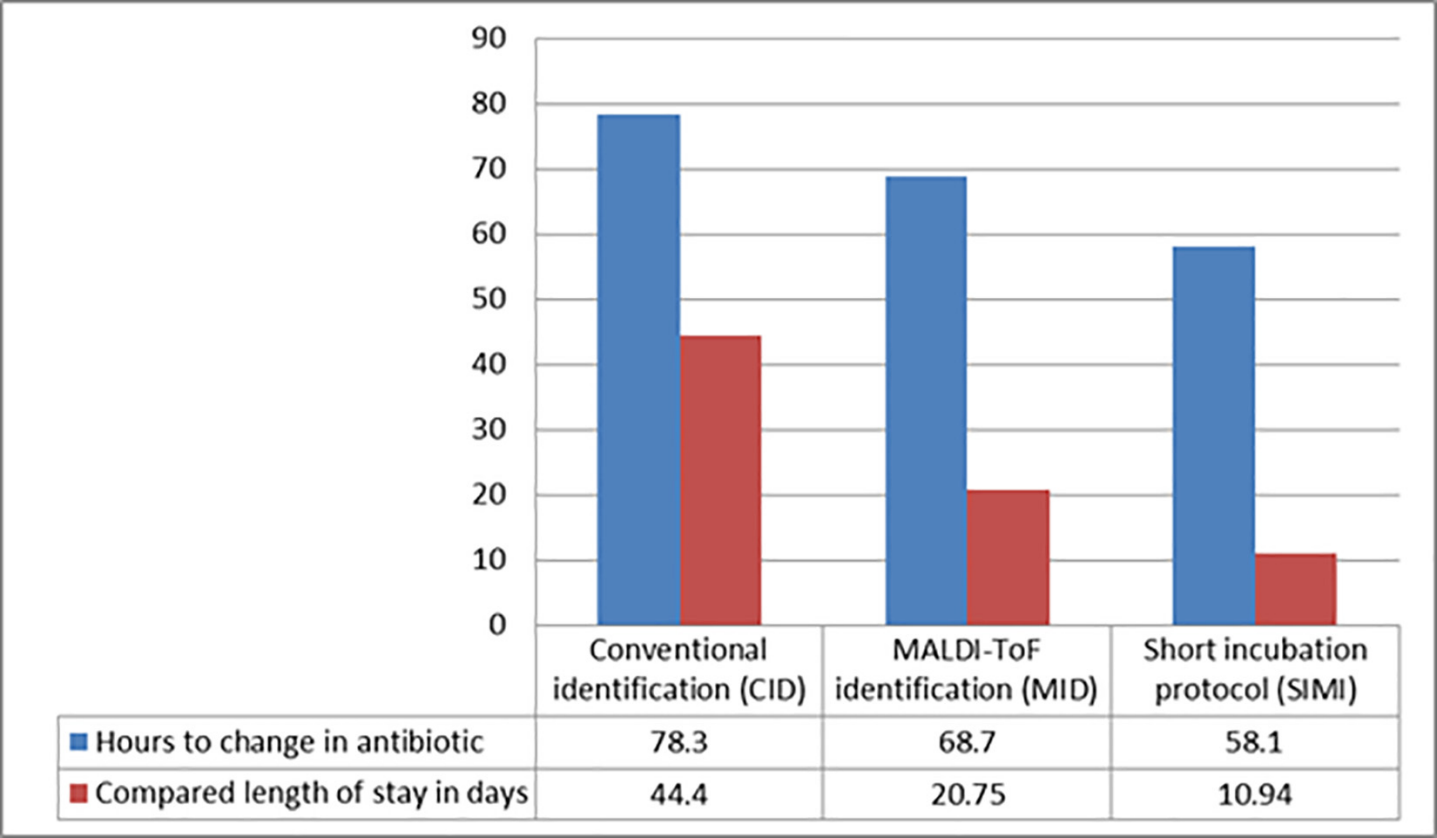
Length of stay of patients after introduction adjustment of the antibiotic. The time to antibiotic change and LOS is compared in the three subgroups of patients that needed their antibiotics to be changed or optimized after the bacterial pathogen was identified from the positive blood culture.

Overall we included and compared 92 patients in our post intervention impact analysis. By assessing the patients that needed change in their antibiotic treatment we could assess the true impact of the expedited identification modalities introduced in 2013 and 2014 (Fig 1). Thirty-one blood cultures were identified that led to potential changes in antibiotics these included duplicates positives obtained from a patient in 2012, and two positive blood cultures obtained from patients in 2014 that was discharged. The obtained isolates from the 2014 duplicates were consistent with contamination and the patients never received antibiotic treatment. Overall twenty eight patients were identified where antibiotic therapy was changed after the blood culture pathogen was resulted; 7 in 2012 (CID), 13 in 2013 (MID) and 8 in 2014 (SIMI). Overall 31.1% patients’ blood culture results were not considered to be optimally treated by their initial empirical antibiotic treatment choice.

**Fig 2:**
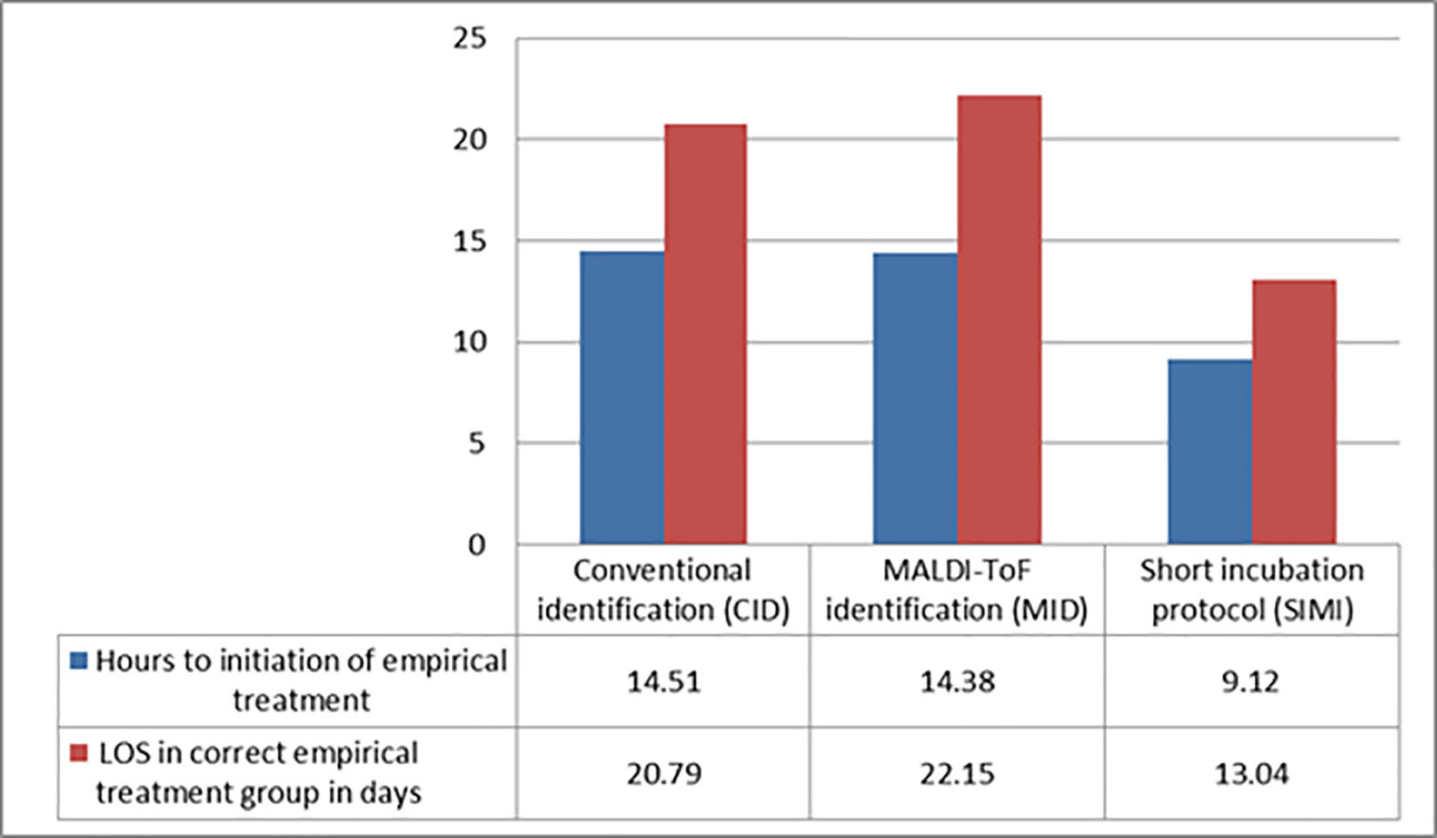
Impact of Early Initiation of the Correct Empirical Therapy.

In Fig 1 the impact on LOS can be seen on the different subgroups, where antibiotics were optimized following the identification of the blood culture pathogen the biggest change in LOS occurred between the CID (2012) and MID (2013) of 53.3% (23.65 days; p = 0.08) with a further 9.82 days (p = 0.06) reduction following introduction of the SIMI, overall this resulted in a statistically significant reduction (p = 0.02) of 75.3% compared to 2012 in this subgroup of patients. The impact of the initiation of early correct empirical therapy was also assessed (Fig 2). The time to initiation of therapy was decreased 36.1% (5.25 hours) overall with an 37.3% associated reduction in LOS of 7.39 days between 2012 and 2014 which was not statistically significant (p = 0.18).

**Fig 3:**
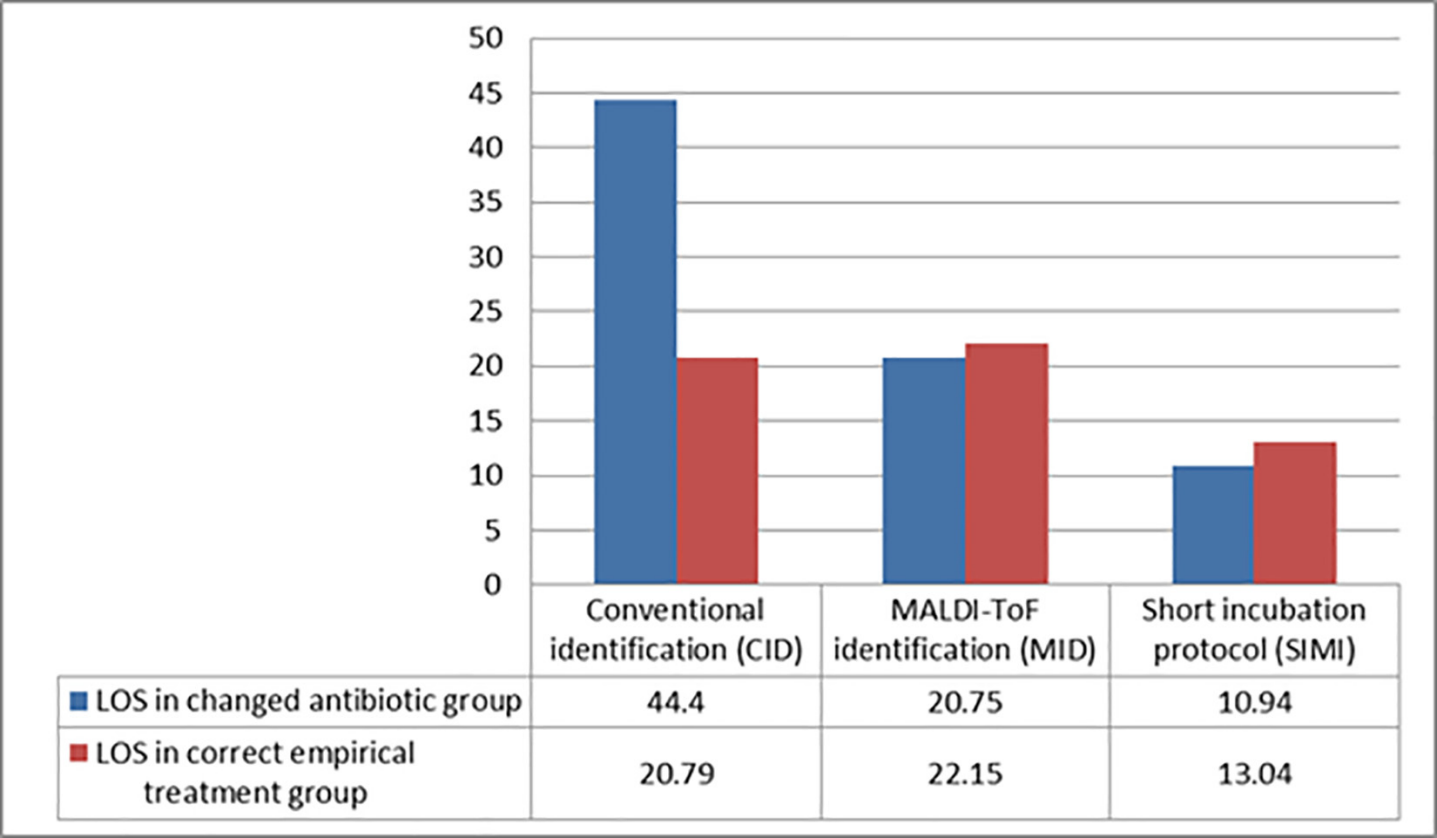
Assessment of the discrepancies in the LOS between the subgroups as a quality of care indicator. The difference in the LOS between the subgroup needing their antibiotic treatment to be changed to the subgroup on the correct empirical therapy serves as a surrogate marker showing that the quality of care has significantly improved after the implementation of the MID and SIMI.

The improved patient outcomes and quality of care in each group was assessed by comparing the group needing antibiotic change to the group which was on the correct empiric antibiotic (Fig 3). There was a significant (p = 0.02) difference in the 2012 group with the group needing change having an overall increased LOS compared to the group needing no change (44.4 days versus 20.79 days), this changed dramatically in 2014 where there was no statistical (p = 0.36) difference in the LOS between the groups needing change and the group where empirical therapy was considered to be optimal (Fig 3). We assessed the mortality risks in the groups, the patient population included in the 2012 group prior to the introduction of any MALDI-ToF identification, had an increased all-cause mortality risk of 1.49 (95% CI: 0.1 to 23.03) and a sepsis attributed mortality risk of 4.34 (95% CI: 0.18 to 103.77) compared to all other groups combined after implementation of MALDI-ToF identification. The 2012 subgroup needing change had all-cause sepsis associated mortality risk 2.88 (95% CI: 0.20 to 40.79) and a direct attributable sepsis related mortality risk 7.67 (95% CI: 0.34 to 171.30) times compared to the same subgroup after implementation of MIDI in 2014.

## Discussion

Sepsis has seen an incremental increase in cases with significant mortality among pediatric patients [5]. Odetola and colleagues reported a reduction in mortality rate from 9.3% to 4.2% with prompt targeted therapy, including appropriate antibiotic and supportive therapy [6, 7]. For improved outcomes, prompt initiation of appropriate antibiotic therapy with the elimination of pathogens is essential. Gram positive bacterial infections are associated with a mortality rate of 52% and Gram negative infections 57% [8, 9]. In a retrospective review of 181 cases of bacteremia, E coli was the most common causative pathogen (42%), followed by group B Streptococcus (23%). Streptococcus pneumoniae was a significant pathogen and was found to be more common in older infants [8, 9].

Our institution services a pediatric population that includes oncology and transplant groups. These patients often have complications such as line associated infections and other hospital acquired infections. To the best of our knowledge this retrospective study is the first in Canada to attempt to measure the impact of rapid identification of bacterial pathogens in a pediatric tertiary care institution. Perez et al. demonstrated a reduction in hospital stay of two days associated with a saving of more than $20,000 per patient in an adult population if pathogens were identified faster and antibiotic treatment optimized earlier [10]. That study, performed in Houston, Texas was limited to an adult population and focused on Gram negative sepsis [10].

In our study we found that the time to empirical antibiotic administration improved by 5.25 hours between 2012 and 2014, the initiation of prompt empirical broad spectrum therapy is supported in several guidelines and documents like the Surviving Sepsis Campaign published in 2012 [2]. Allthough a reduction in time was seen to the Gram stain result between 2012 and 2014 of 15% we could not find a statistical or clinical benefit associated with the earlier expedited result. No empirical antibiotic regimens were adjusted based on the Gram stain results obtained earlier. The reduction in the average Gram stain result occurred after the laboratory service hours were extended in 2013.

The institution of MALDI-TOF identification led to a significant decrease in time to identify a specific pathogen, and a short incubation MALDI-TOF identification protocol from positive blood cultures further reduced time to identification by 38.78 hours overall (p = 7.46E-07) and 17.75 hours (30.4%) comparing SIMI to MID. In the subgroup where antibiotics needed to be changed optimization of antibiotic therapy occurred 20.2 hours (25.8%) earlier compared to 2012 when conventional methods to identification were used. This finding is consistent with a study by Kohlmann et al. where the earlier identification of Gram negative pathogens led to optimization of treatment in 51% of cases [11]. A reduction in length of stay was noted between the CID group and the MID group of 2.1 days. The most significant reduction was between the MID and SIMI groups. The LOS in the SIMI group was reduced from 21.7 days to 12.28 days (p = 0.09) representing a 43.5% reduction overall in LOS (Tables 1 and 2).

The earlier initiation of antibiotics certainly contributed to the overall improvement in outcomes in patients with sepsis and bacteremia, we did not find a similar association when the expedited Gram stain results were reviewed. During our assessment of the impact in this subgroup there was an overall reduction in the initiation of the empirical antibiotics of 36.1% (p = 0.14) and LOS of 37.3% (p = 0.18) although these were found not to be statistically significant, it is hard to ignore the clinical impact of this finding as it was certainly partly supportive for the overall reduction in LOS of the three groups, this however did not explain the change in LOS of the subgroups needing change to their antibiotic therapy.

The patient subgroup of interest was the groups in each year where the antibiotic needed to be changed following the identification of the pathogen from the positive blood cultures. Our findings demonstrates that optimization of antimicrobial therapy and switching to more appropriate therapy occurred earlier. By evaluating the subgroup of patients where antibiotics were changed after identification of the pathogen, it was clear that the MID reduced the time to initial identification and patients received targeted therapy 20.2 hours earlier overall with a further reduction in LOS seen after implementation of the rapid short incubation protocol (Table 1 and Fig 1). We further evaluated the validity of our findings by comparing the differences in LOS in each subgroup between the group where antibiotics were changed and the group where empirical antibiotics were considered to be optimal to show if there was an improvement in care (Fig 3). This was statistically evaluated to the effect as a quality of care indicator; it was clear that the discrepancy in LOS between the group needing change in their antibiotic therapy and the group where empirical therapy was optimal improved. In the 2012 group there was significant increase in the average LOS of stay of 23.61 days (p = 0.02) in the group needing change compared to the optimal empirical therapy group. The quality of care improved in 2013 and 2014 following the implementation of the routine MID and the SIMI and there was no statistical significant differences observed in outcomes as measured by LOS (Fig 3). This finding was quite significant as it clearly indicated that not only did the overall LOS improve but the overall quality of care had substantially been improved based on the earlier identification of the pathogen. This was also overall associated with a fourfold reduction in mortality risk associated with bacteremia and a more than seven fold reduced risk of sepsis related mortality in patients with sepsis or bacteremia where antibiotics needed to be changed based on pathogen identification after implementation of the MID and the SIMI compared to conventional diagnostic methods.

Several factors could however be responsible for the improved LOS. The factors that appeared to be most significant therefore were earlier initiation of empiric antibiotics and earlier identification of the infecting pathogen with targeted antibiotic therapy initiated earlier in the MID (2013) and SIMI (2014) groups. Although the 5.38 hours reduction in time to earlier initiation of treatment between 2012 and 2014 was not statistically significant (P = 0.14) it is difficult to ignore the fact that this was likely associated with a downstream effect in reducing LOS in the subgroup of patients where no change in antibiotic coverage was required.

Limitations of this study include the relatively small sample size and the heterogenicity of the patient population and collected data however the results are clinically extremely relevant. The current concept in the treatment of bacteremia and septicemia heavily relies on the early initiation of the correct empirical therapy our study however assessed the group of this patient population where the empirical antibiotic therapy initiated was not effective in covering the offending pathogen, this partly is responsible for the relatively small subgroup of patients. Another potential confounding factor that could influence the data is the difference in bacterial pathogens among the different groups, Perez *et al*. demonstrated a significant saving related to the earlier identification of Gram negative pathogens [10] with MALDI-ToF however considering the selection bias in this study we attempted to evaluate impact on all patients outcomes where antibiotic needed to be changed after identification of the pathogen. Contrary to other studies like Verroken *et al*. we tried to assess the impact per patient and not per test, this resulted in a relatively small heterogenic study population but with the benefit of assessing the holistic impact on patient outcomes. We conclude that rapid identification of bacterial pathogens in pediatric blood cultures with a rapid incubation MALDI-TOF identification protocol contributes to earlier optimization of antibiotic therapy playing an important role in reducing the length of hospitalization and is associated with a reduced mortality risk due to bacteremia and sepsis complications. Rapid identification of pathogens from blood cultures should become standard of care in complicated pediatric hospital care settings.

## Supporting Information

S1 Table Revised Statistical Analysis. (XLSX)

S1 Waiver Research and Ethics Board. (PDF)
