# Supplementary material for: Quality of Care Is Improved by Rapid Short Incubation MALDI-ToF Identification from Blood Cultures as Measured by Reduced Length of Stay and Patient Outcomes as Part of a Multi-Disciplinary Approach to Bacteremia in Pediatric Patients
Source: PLoS One. 2016 Aug 11;11(8):e0160618. doi: 10.1371/journal.pone.0160618 (PMC4981314; doi:10.1371/journal.pone.0160618)
Supplement: S1 Waiver — (PDF) [file pone.0160618.s002.pdf]

Western Research

Research Ethics

April 24, 2015

Dr. Johan Delport
Schulich School of Medicine and Dentistry
800 Commissioners Rd. E
Victoria Hospital
London, ON
Room B10-105

Dear Dr. Delport,

Re: Quality improvement initiatives: Division of Microbiology, REB 106659

The Chair has reviewed the above-referenced project and it does not require approval of a Research Ethics Board. In accordance with the Tri-Council Policy Statement 2: Ethical Conduct of Research Involving Humans, Article 2.5. "Quality assurance and quality improvement studies, program evaluation activities, and performance reviews, or testing within normal educational requirements when used exclusively for assessment, management or improvement purposes, do not constitute research for the purposes of this Policy, and do not fall within the scope of REB review".

In my opinion, the above-referenced research project falls within that description.

I wish you the best of luck with your work.

Most sincerely,

Vikki Tran
Ethics Officer, Office of Research Ethics
Western University
1393 Western Road, Support Services Building
London, ON, Canada
e. vikki.tran@uwo.ca
p. 519.661.2111 (80242)
f. 519.850.2466

cc:file
